# LATE‐LIFE EDUCATION IMPROVES ADULT EXECUTIVE FUNCTION: THE PROAME TRIAL

**DOI:** 10.1002/alz.086705

**Published:** 2025-01-09

**Authors:** Clarisse Vasconcelos Friedlaender, João Victor de Faria Rocha, Kelle Luisa dos Santos Tomaz, Emma Patrice Ruppert, Aida Lourandes, Ana Paula Zacarias, Joanna de Castro Magalhães Assenção, Luciana Paula Rincon, Norton Gray Ferreira Ribeiro, Howard J. Rosen, Lea T. Grinberg, Francisca Izabel Pereira Maciel, Paulo Caramelli, Elisa de Paula França Resende

**Affiliations:** ^1^ Universidade Federal de Minas Gerais, Belo Horizonte, Minas Gerais Brazil; ^2^ Faculdade de Medicina de Ciências Médicas de Minas Gerais, Belo Horizonte, Minas Gerais Brazil; ^3^ Escola Municipal Dr Julio Faria, Belo Horizonte Brazil; ^4^ Global Brain Health Institute, University of California, San Francisco, CA USA; ^5^ UCSF Weill Institute for Neurosciences, University of California, San Francisco, CA USA; ^6^ Weill Institute for Neurosciences, University of California San Francisco, San Francisco, CA USA; ^7^ Global Brain Health Institute, University of California San Francisco, San Francisco, CA USA

## Abstract

**Background:**

Education is a recognized modifiable dementia risk factor. To boost cognitive reserve and reduce dementia risk in Brazil’s vulnerable populations, we conceived a literacy program (PROAME trial) targeting low‐educated adults, aiming to explore how executive function and individual differences influence program effectiveness.

**Method:**

We screened 130 adults, with 108 meeting enrollment criteria. They were allocated in control (CG, n = 51) and intervention (IG, n = 57) groups, according to residence area. Subjects underwent a comprehensive neuropsychological assessment at baseline and after six months, covering global cognition, executive function, memory, and language.

**Result:**

We excluded 28 participants for incomplete post‐intervention assessments. 77 completed all procedures, with 62 (CG = 26; IG = 36) finishing all executive function tasks. Mean age = 59.16 years (±9.22), mean education = 2.21 years (±2.20), with 62.3% mixed‐race, 29.5% Black, and 75.8% female. Mini‐Mental State Examination mean score = 22.08 (±2.74). We created an executive function composite score (EFCS) including phonemic verbal fluency, reverse digit span, and rapid naming. Comparing EFCS pre‐ and post‐ intervention using a linear regression, participants (in both groups) with higher baseline EFCS showed less post‐intervention improvement (ANCOVA; F = 6.233; p<0.001). IG displayed significant EFCS enhancement compared to CG (p = 0.003). Considering possible adult illiteracy causes, participants who reported learning difficulties exhibited the lowest EFCS at baseline, compared to rural residence, lack of family support, and childhood labor groups.

**Conclusion:**

The literacy program positively impacted executive function. IG exhibited significant EFCS improvement after six months, demonstrating late formal education acquisition as an effective cognition‐enhancing strategy. Notably, those with lower initial executive function levels experienced greater improvements, suggesting enhanced benefits for those initially lacking executive skills. However, the learning difficulties group derived lesser benefits from the intervention, highlighting the importance of considering individual differences. Childhood learning difficulties may hint at undiagnosed developmental disorders, influencing cognitive reserve development early on. Recognizing the pivotal role of executive function in enhancing cognitive reserve, promoting this domain through literacy‐focused interventions can be a valuable tool in preventing cognitive decline and dementia.

